# Selumetinib Activity in Thyroid Cancer Cells: Modulation of Sodium Iodide Symporter and Associated miRNAs

**DOI:** 10.3390/ijms19072077

**Published:** 2018-07-17

**Authors:** Sabine Wächter, Annette Wunderlich, Brandon H. Greene, Silvia Roth, Moritz Elxnat, Sebastian A. Fellinger, Frederik A. Verburg, Markus Luster, Detlef K. Bartsch, Pietro Di Fazio

**Affiliations:** 1 Department of Visceral Thoracic and Vascular Surgery, Philipps-University Marburg, Baldingerstrasse, 35043 Marburg, Germany; wunderli.an@gmail.com (A.W.); rothsi@med.uni-marburg.de (S.R.); moritz_elxnat@gmx.de (M.E.); bartsch@med.uni-marburg.de (D.K.B.); 2 Institute of Medical Biometry and Epidemiology, Philipps-University Marburg, Bunsenstrasse 3, 35037 Marburg, Germany; brandon.greene@staff.uni-marburg.de; 3 Department of Nuclear Medicine, Philipps-University Marburg, Baldingerstrasse, 35043 Marburg, Germany; sebastian.fellinger@med.uni-marburg.de (S.A.F.); FrederikAnton.Verburg@uk-gm.de (F.A.V.); luster@med.uni-marburg.de (M.L.)

**Keywords:** radioiodine refractory differentiated thyroid cancer, miRNAs, MEK (mitogen-activated protein kinase kinase) inhibitor

## Abstract

Background: The MEK (mitogen-activated protein kinase)–inhibitor selumetinib led to increased radioiodine uptake and retention in a subgroup of patients suffering from radioiodine refractory differentiated thyroid cancer (RR-DTC). We aimed to analyse the effect of selumetinib on the expression of sodium iodide symporter (NIS; SLC5A5) and associated miRNAs in thyroid cancer cells. Methods: Cytotoxicity was assessed by viability assay in TPC1, BCPAP, C643 and 8505C thyroid cancer cell lines. NIS, hsa-let-7f-5p, hsa-miR-146b-5p, and hsa-miR-146b-3p expression was determined by quantitative RT-PCR. NIS protein was detected by Western blot. Radioiodine uptake was performed with a Gamma counter. Results: Selumetinib caused a significant reduction of cell viability in all thyroid cancer cell lines. NIS transcript was restored by selumetinib in all cell lines. Its protein level was found up-regulated in TPC1 and BCPAP cells and down-regulated in C643 and 8505C cells after treatment with selumetinib. Treatment with selumetinib caused a down-regulation of hsa-let-7f-5p, hsa-miR-146b-5p and hsa-miR-146b-3p in TPC1 and BCPAP cells. In 8505C cells, a stable or down-regulated hsa-miR-146b-5p was detected after 1h and 48h of treatment. C643 cells showed stable or up-regulated hsa-let-7f-5p, hsa-miR-146b-5p and hsa-miR-146b-3p. Selumetinib treatment caused an increase of radioiodine uptake, which was significant in TPC1 cells. Conclusions: The study shows for the first time that selumetinib restores NIS by the inhibition of its related targeting miRNAs. Further studies are needed to clarify the exact mechanism activated by hsa-miR-146b-5p, hsa-miR-146b-3p and hsa-let7f-5p to stabilise NIS. Restoration of NIS could represent a milestone for the treatment of advanced RR-DTC.

## 1. Introduction

Differentiated thyroid carcinoma (DTC), including papillary thyroid carcinoma (PTC) and follicular thyroid carcinoma (FTC), accounts for ~93% of thyroid carcinomas (TC) and has a favourable prognosis [1]. However, approximately 5% of patients with DTC show aggressive metastatic disease, no response to radioiodine therapy [2], and are associated with a poor prognosis [3]. Similarly, patients suffering from anaplastic thyroid carcinoma (ATC), which accounts only for 2% of thyroid carcinomas, have a mortality rate higher than 90% [4,5,6].

Effective treatment strategies to overcome the fatal prognosis of these radioiodine refractory thyroid cancers (RR-DTC) are still lacking, and new therapeutic strategies are urgently needed [7].

The mutation and/or loss of function of genes encoding for proteins stably expressed in thyroid tissue cause the loss of radioiodine avidity, leading to an inefficacious radioiodine therapy as confirmed by in vitro studies [8,9]. In particular, dysfunction of sodium iodide symporter (NIS) is based on the suppression or loss of the *NIS* gene and/or decreased migration and localisation of its protein at the cell membrane surface [10]. Therefore, new substances were developed to promote the restoration of the Na^+^/I^−^ symporter (NIS) and increase radioiodine storage. In a small study of 20 patients suffering from RR-DTC, it has been shown that the MEK (mitogen-activated protein kinase)–inhibitor selumetinib led to increased radioiodine uptake and retention [11].

Moreover, microRNAs (miRNAs, miRs) regulate gene expression by binding to their target mRNAs and blocking their translation. Beneath their relevance as diagnostic and prognostic factors [12], miRNAs have emerged as a promising therapeutic target in many diseases including thyroid cancer [13,14]. OncomiR hsa-miR-146b—especially hsa-miR-146b-5p—is significantly over-expressed in PTC and associated with tumor migration, invasion, EMT (epithelial-mesenchymal transition) and resistance to chemotherapeutics [14,15,16,17]. The over-expression of miRNA hsa-miR-146b-5p is promoted by RET/PTC3 (REarranged during Transfection) and BRAF (v-Raf murine sarcoma viral oncogene homolog B) activation [15]. It is inversely correlated with NIS expression [18] due to its high affinity for the 3′UTR (3′untranslated region) of NIS mRNA [14]. In silico analysis revealed NIS as target of hsa-let-7f-5p [19] that belongs to the let-7 family of tumor suppressor miRNAs. The deregulation/suppression of let-7 family members acts in several types of cancer [20], including DTC [21,22,23]. Interestingly, some histopathological subgroups of DTC have shown a stable or up-regulated expression of them [19]. Yet, little is known regarding the distinct function of let-7 in DTC. Among them, hsa let-7f is described as critical for the proper regulation of growth and differentiation of thyroid cells. In particular, hsa-let-7f-5p was reported to exert its tumor suppressor role by reducing cell proliferation and inducing thyroid differentiation markers [24].

In this study, we aimed to analyse the efficacy of selumetinib in different thyroid carcinoma cell lines. In particular, we aimed to evaluate the modulation of NIS and associated miRNAs mediated by selumetinib.

## 2. Results

### 2.1. Selumetinib Cytotoxic Effects

**Figure 1:**
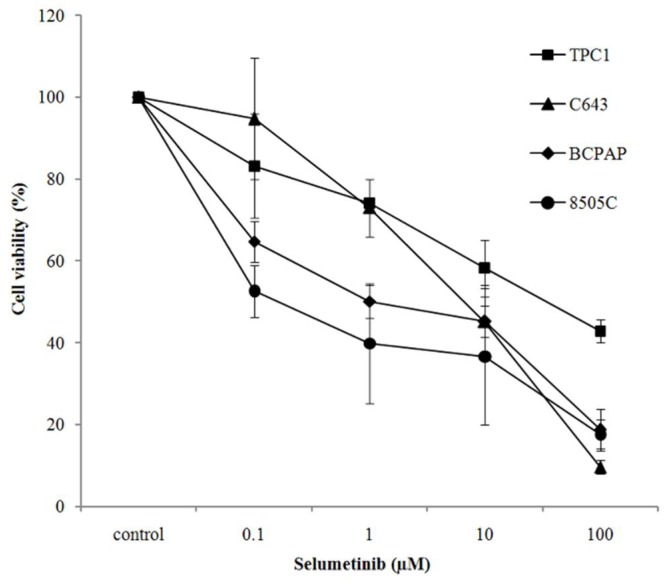
Selumetinib effect on cell viability. Cell viability of TPC1, C643, BCPAP and 8505C cells treated with an increasing concentration of selumetinib for 144 h. Cell viability is expressed relative to the untreated control, which was set to 100%. Data represent mean ± SD of three experiments performed in triplicates.

Selumetinib exerted a cytotoxic effect in TPC1, C643, BCPAP and 8505C thyroid cancer cell lines. Interestingly, BCPAP and 8505C cells, both carrying a BRAFV600E mutation, were more sensitive to the drug than C643 and TPC1 cells. They showed a significant reduction of cell viability already at concentrations as low as 0.1 and 1 µM, as shown here below after 144 h of treatment (Figure 1 and Table 1).

**Figure 2:**
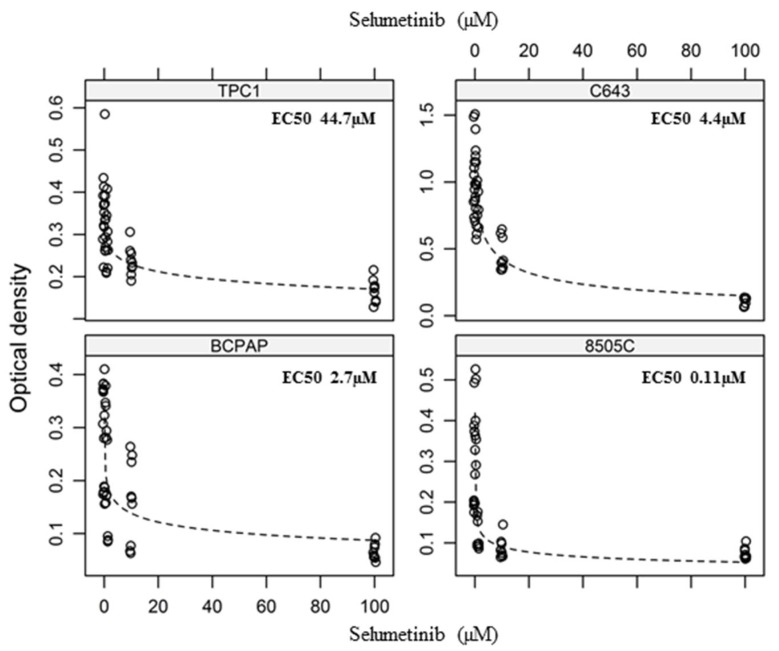
Dose-response to selumetinib. Curves of the four cell lines treated with increasing concentration of selumetinib for 144 h. Results of three independent experiments performed in triplicates (detailed data c.f. Table 1). *p* < 0.05 compared to control (detailed *p*-values c.f. Table 1).

Raw data were further analysed by modelling a dose-response curve, which allowed us to determine the EC50 value for each cell line (Figure 2). Thus, the sensitivity to selumetinib, expressed as EC50, was most pronounced in 8505C cells that were characterised by an EC50 value of 110 ± 50 nM after 144 h. For C643 cells and BCPAP cells, comparable effects were achieved at low micro molar concentrations (EC50 2.8 ± 3.3 and 4.4 ± 1.4 µM). TPC1 cells were found to be less sensitive to selumetinib treatment (EC50 44.7 ± 36.6 µM) in our settings (Figure 2, Table 1).

In conclusion, cells carrying a BRAFV600E mutation were found to be more sensitive at lower drug concentrations (100 nM) than those lacking this mutation.

### 2.2. NIS Restoration after Selumetinib Treatment

**Figure 3:**
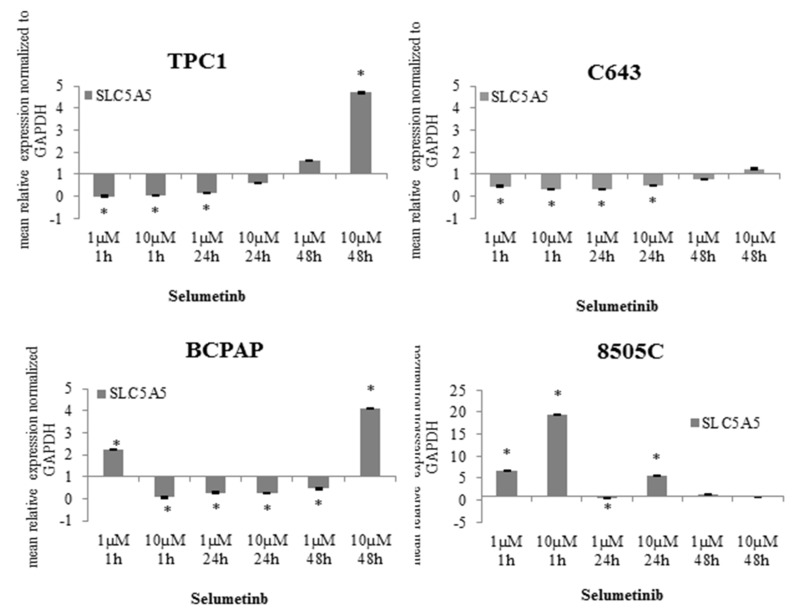
SLC5A5 (sodium iodide symporter (NIS)) expression affected by selumetinib. RT-qPCR of SLC5A5 transcript in TPC1, C643, BCPAP and 8505C cells treated with 1 and 10 µM selumetinib. SLC5A5 transcript was normalized to GAPDH (Glyceraldehyde 3-Phosphate Dehydrogenase). Results are expressed relative to the untreated control. Data represent mean ± SEM of experiments performed in triplicates. * *p* < 0.05 regarded as significant.

It is well known that NIS expression and its function is linked to a better outcome for patients affected by advanced thyroid cancer [25]. Here, the expression of NIS transcript was analysed after treating the cells with 1 µM and 10 µM selumetinib for up to 48 h. In TPC1 cells, NIS transcript was not modulated after short-time treatment (1 h). Interestingly, it could be observed that long-time treatment (48 h) determined a significant up-regulation of its level (4.7 fold), especially with the higher selumetinib concentration (10 µM) (Figure 3). NIS transcript was also detectable in C643 cells and its expression was restored to a basal level after 48 h treatment with selumetinib (Figure 3). In BCPAP cells, NIS transcript was up-regulated (4.1 fold) by prolonged treatment with 10 µM selumetinib (Figure 3). 8505C cells were shown to be the best candidate for selumetinib-dependent stimulation of NIS up-regulation. The treatment with the inhibitor caused a significant over-expression of NIS transcript in these cells even after 1 h. The level of NIS was still stable after 48 h of treatment (Figure 3).

To better understand the modality of action of selumetinib, TPC1, C643, BCPAP and 8505C cells were pre-treated for one hour with 10 µg/mL actinomycin, a potent transcription inhibitor, and then incubated with 10 µM selumetinib for 48 h.

**Figure 4:**
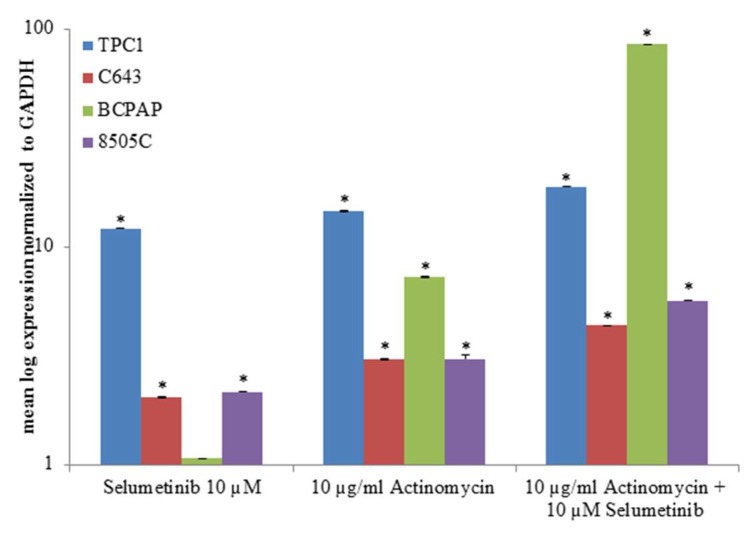
NIS transcript expression after treatment with actinomycin and selumetinib. TPC1, C643, BCPAP and 8505C cells were pre-treated with 10 µg/mL actinomycin for one hour before adding 10 µM selumetinib for 48 h. SLC5A5 was normalized to GAPDH. Results are expressed relative to the untreated control. Data represent mean ± SEM of experiments performed in triplicates. * *p* < 0.05 regarded as significant.

As shown in Figure 4, SLC5A5 transcript increased after actinomycin treatment; actinomycin had an additive effect to the one exerted by selumetinib in all cell lines.

In summary, the *NIS* gene transcript was restored by selumetinib in all cell lines used for this study (Table 2).

The efficacy of selumetinib has shown a potent induction after short-time treatment in 8505C only, whereas similar effects could be observed in the other three cell lines after 48 h treatment. This effect does not correlate with BRAF mutation status.

### 2.3. Selumetinib Impaired Expression of NIS Related miRNAs

**Figure 5:**
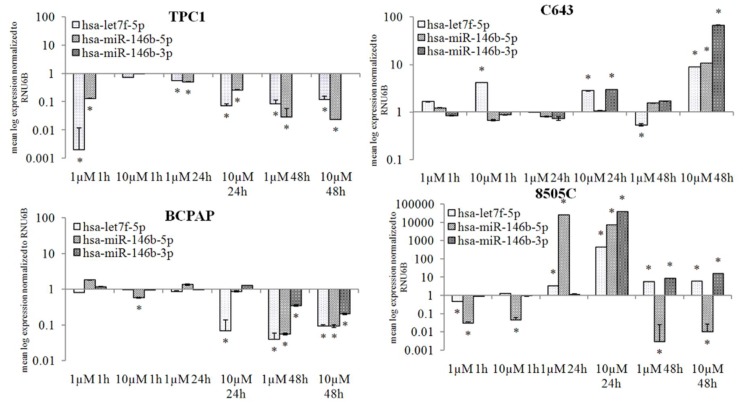
MiRNA expression affected by selumetinib. RT-qPCR of hsa-let7f-5p, hsa-miR-146b-5p and, hsa-miR-146b-5p in TPC1, C643, BCPAP and 8505C cells treated for 48 h with 1 and 10 µM selumetinib. MiRNA transcripts were normalized to RNU6B. Results are expressed relative to the untreated control. Data represent mean ± SEM of experiments performed in triplicates. * *p* < 0.05 regarded as significant.

Based on our previous study, the expression of has-let7f-5p, considered to be involved in the NIS regulation, was analysed after treatment with 1 and 10 µM selumetinib. Furthermore, two other miRNAs, that based on previous studies and in silico analysis are predictably targeting SLC5A5, were analysed. Selumetinib induced a significant suppression of hsa-let7f-5p and hsa-miR-146b-5p in TPC1 cells. Hsa-miR-146b-3p was not detectable, neither in the untreated control nor in the selumetinib treated samples (Figure 5).

In C643 cells, hsa-let7f-5p was up-regulated at all time-points of treatment with selumetinib, and its over-expression was significant in cells treated with 10 µM (up to 9-fold). Hsa-miR-146b-5p showed a stable expression; its level was up-regulated after 48 h treatment (about 10-fold). Hsa-miR-146b-3p was significantly up-regulated after 24 h (3-fold) and 48 h (about 67-fold) treatment with 10 µM selumetinib (Figure 5).

In BCPAP cells, only a prolonged treatment (48 h) with selumetinib caused a significant down-regulation of all miRNAs (Figure 5).

In 8505C cells, selumetinib caused a stable down-regulation of hsa-miR-146b-5p. The other two miRNAs hsa-let7f-5p and hsa-miR-146b-3p showed a general stable expression and a significant up-regulation after 24 h of treatment (Figure 5).

Taken together, our data clearly demonstrate that selumetinib treatment affects miRNAs expression. The selected miRNAs were generally suppressed in TPC1 and BCPAP cells. In C643 and 8505C cells, however, selumetinib led to a stable and/or increased level of these miRNAs, with exception of hsa-miR-146b-5p, which was suppressed in 8505C cells after 48 h (Table 1).

### 2.4. miRNAs Expression Correlation with the Validated Target NIS after Selumetinib Administration

**Figure 6:**
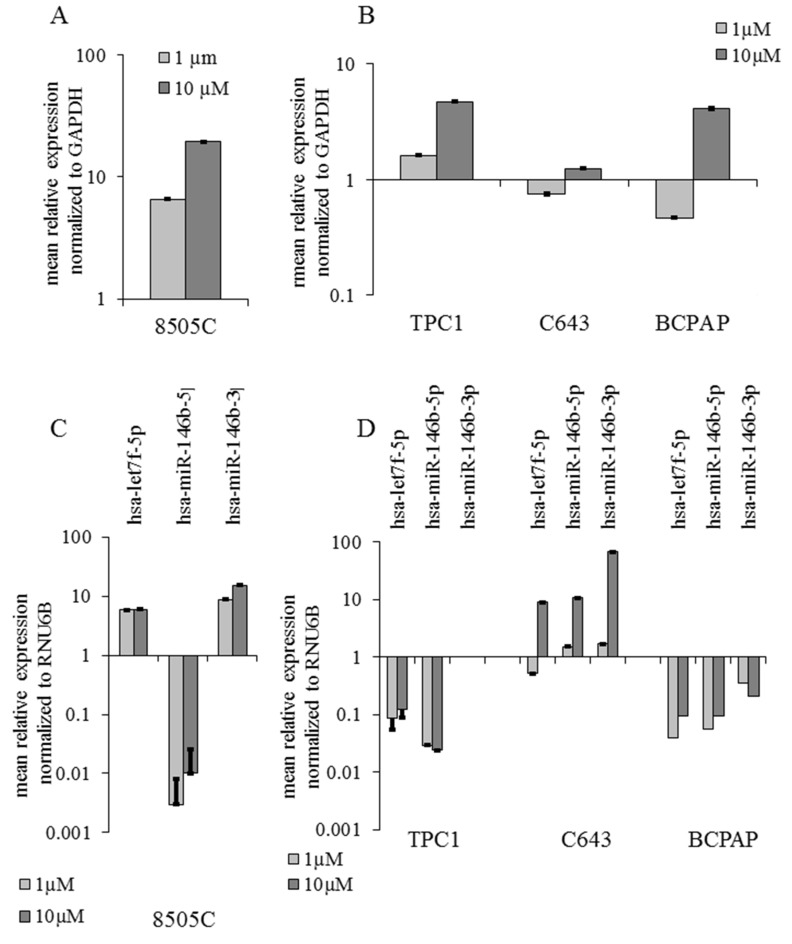
Summary of selumetinib mediated effects on NIS (SLC5A5) and miRNA expression. RT-qPCR of SLC5A5, hsa-let7f-5p, hsa-miR-146b-5p and hsa-miR-146b-3p transcripts in 8505C cells (**A**,**C**) treated with selumetinib for 1 h and TPC1 C643 and BCPAP cells (**B**,**D**) treated for 48 h. SLC5A5 was normalized to GAPDH (Glyceraldehyde 3-Phosphate dehydrogenase). MiRNAs were normalized to RNU6B. Results are expressed relative to the untreated control. Data represent mean ± SEM of experiments performed in triplicates.

Comparing the data concerning selumetinib-mediated effects on the expression of NIS transcript and NIS related miRNAs, an inverse correlation between these two parameters could be observed. As documented here in Figure 6, NIS transcript over-expression and miRNA down-regulation were observed in TPC1 and BCPAP cells; C643 cells, characterized by the over-expression of all three analysed miRNAs, showed only a stable expression of NIS that could be attributed to a stabilization of its transcript mediated by miRNAs (Figure 6B–D). In 8505C cells, hsa-miR-146b-5p showed a significant down-regulation whereas hsa-let7-5p and hsa-miR-146b-3p were over-expressed, thus confirming an inverse correlation between the up-regulated status of NIS and the down-regulation of hsa-miR-146b-5p (Figure 6A–C). 

### 2.5. Selumetinib Treatment Did Not Always Change NIS Protein Expression

**Figure 7:**
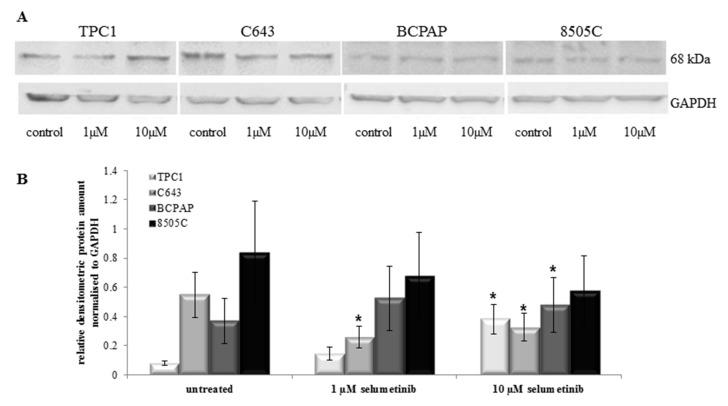
Selumetinib effect on NIS protein level. (**A**) Western blotting analysis (SDS-Page 4–12%, 50 µg protein/lane) of NIS protein in TPC1, C643, BCPAP and 8505C cells treated with selumetinib at indicated concentrations and probed with an anti-NIS antibody (clone *FP5A*). Human GAPDH was used as an equal loading control; (**B**) densitometry of Western blot bands of NIS normalised vs. GAPDH. Means ± SEM are shown. * *p* < 0.05 regarded as significant.

NIS function is important for the response to radioiodine therapy in patients affected by thyroid cancer, and therefore its expression represents a predictive biomarker for the sensibility of those patients going to be treated with radioiodine. Here, NIS protein expression was analysed by Western blotting using a monoclonal NIS antibody (clone *FP5A*). Treatment with selumetinib caused an increase of NIS protein level in TPC1 cells, as shown in Figure 7A.

In particular, 10 µM selumetinib caused a significant over-expression of NIS protein in TPC1 cells, confirmed by densitometric analysis (Figure 7B). NIS over-expression was observed in BCPAP also, especially after treatment with 10 µM selumetinib. C643 and 8505C cells showed a decrease of NIS protein after treatment with 1 and 10 µM selumetinib. The down-regulation was significant in C643 only (Figure 7A,B).

In conclusion, NIS protein was found to be modulated in all cell lines after treatment with selumetinib. TPC1 and BCPAP cells showed an up-regulation, whereas C643 and 8505C cell lines were characterized by a down-regulation of NIS protein after treatment with selumetinib.

### 2.6. Sodium Iodide Symporter Activity after Selumetinib Treatment

The sensitivity to radioiodine uptake is relevant clinical data for the diagnosis of thyroid malignancies and their treatment. To further define the mechanism of action of selumetinib, TPC1, BCPAP, C643 and 8505C cells, previously treated with 10 µM selumetinib, were incubated with I-131 and the radioactivity was measured on a gamma counter.

**Figure 8:**
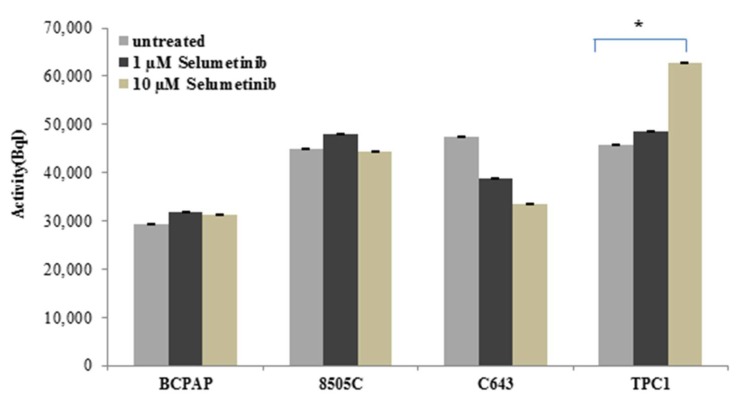
Radioiodine uptake in thyroid carcinoma (TC) cells after treatment with selumetinib. BCPAP, 8505C, C643 and TPC1 cell lines were treated for 48 h with 10 µM selumetinib and 1 MBql I-13. The radioactivity of cells was measured on a gamma counter. Data represent mean ± SEM of experiments performed in triplicates. * *p* < 0.05 regarded as significant.

As shown in Figure 8, 10 µM selumetinib caused a significant increase of the radioiodine uptake in TPC1 cells, whereas in BCPAP and 8505C cells the activity was almost stable or not significantly up-regulated. C643 cells were characterized by a reduction of radioiodine uptake.

In conclusion, we could observe that TPC1 cells, which are less sensitive to treatment with selumetinib, showed an increase of radioiodine uptake. The other cell lines showed a basal activity which was more or less pronounced after treatment with selumetinib.

## 3. Discussion

This study first focused on the cytotoxic activity of selumetinib in different thyroid cancer cell lines that differ in HRAS, BRAF and TP53 mutation status. Among them, the cell line 8505C, carrying a BRAFV600E mutation, was found the most sensitive to selumetinib cytotoxicity. This corresponds to the observation that human TC cells carrying BRAFV600E mutations are preferentially sensitive to MEK inhibitors [26], which was confirmed in preclinical models also [27]. The lower sensitivity of BCPAP, also carrying a BRAFV600E mutation, could be attributed to an additional mutation of TP53 (inactivating, Asp259Tyr) [28], which leads to aberrant activation of the RAS/RAF/MEK/ERK pathway and may contribute to chemo-resistance [29,30].

From two cell lines carrying wild type BRAF, the cell line C643 was as sensitive as BCPAP. This result is in line with the observation of Liu et al., who found C643 cells harbouring an HRAS (GTPase HRAS; transformin protein p21) mutation that confers sensitivity to MEK inhibitors similar to those cells with mutated BRAF [31]. TPC1 cells were less sensitive to selumetinib administration. Presumably, this is due to the fact that these cells harbour a Ret/PTC1 rearrangement that confers a resistance to the anti-proliferative effects exerted by MEK inhibition to these cells [31]. Our study furthermore confirmed the inhibitory effect on MEK exerted by selumetinib, as was previously published [26,32]. Selumetinib is currently used in therapy of patients affected by solid cancer, and it has shown a high affinity in patients suffering from non-small cell lung cancer (NSCLC) [33], melanoma [34] and breast cancer [35]. Additionally, in vitro studies have shown that selumetinib is capable of blocking cell proliferation and metastasis in several solid cancer models [36,37,38].

This study focused on the effect of selumetinib on NIS transcript and protein expression as well as on a subset of miRNAs that potentially target NIS mRNA. As documented above, three of the four cell lines respond to selumetinib with restoration of the NIS transcript, albeit with different modulation patterns and expression levels. Thus, our results confirm the observation that the suppression of MAPK (mitogen-activated protein kinase) signaling (via MEK inhibition) contributes to the re-differentiation of TC in terms of the re-expression of thyroid-specific genes, as previously published [11]. Moreover, it was shown here for the first time that MEK inhibition mediated by selumetinib leads to stabilised NIS-transcript expression in vitro. Moreover, treatment with transcriptional inhibitor actinomycin promoted the expression of NIS transcript, and its effect was additive to selumetinib treatment, thus confirming that the MEK inhibitor stabilised NIS transcript, offering a higher amount of NIS transcript pool to the treated cells that could probably act more efficiently in the turn-over of the sodium iodide symporter.

In addition to the restoration of NIS transcript observed in all cell lines, a modulation of NIS protein level was found in all cell lines treated with selumetinib. The 68 kDa fragment representing non-/partial-glycosylated NIS [39] was up-regulated in TPC1 and BCPAP cells and down-regulated in C643 and 8505C cells.

The stable radioiodine uptake activity confirmed the functionality of the sodium iodide symporter. Interestingly, TPC1 cells, which are less sensitive to the cytotoxic activity of selumetinib, showed a significant increase of radioiodine uptake and NIS protein that could be attributed to the absence of hsa-miR-146b-3p expression in these cells. The other three cell lines—BCPAP, C643 and 8505C—included in the study, even if at low levels, still showed an expression of hsa-miR-146b-3p. The presence of has-miR-146b-3p could mitigate the effect of selumetinib by neutralizing the increase of the radioiodine uptake and expression of NIS protein.

It has been shown that NIS is under the control of epigenetic mechanisms [40,41,42]. Our findings revealed that selumetinib treatment affects the expression of miRNAs that selectively target NIS. The selected miRNAs hsa-let7f-5p, hsa-miR-146b-5p and hsa-miR-146b-3p were suppressed in TPC1 and BCPAP cells, both derived from PTC (carrying p53wt) and in part in 8505C cells (mutated p53), derived from a PDTC. In contrast, the expression level of these miRNAs was increased in C643 cells (ATC), where NIS gene expression was not significantly affected.

The study here proposed showed an existing inverse correlation between the three miRNAs hsa-let7f-5p, hsa-miR-146b-5p and hsa-miR-146b-3p and their target NIS in TPC1 and BCPAP cells. This might lead to the conclusion that selumetinib contributes to NIS-induction via the suppression of its related miRNAs, thus supporting the previous study of Li et al. showing that hsa-miR-146b could regulate NIS expression/activity and that its suppression can restore the radioiodine-sensitivity in dedifferentiated cells [14]. As we found, hsa-miR-146b (especially hsa-miR-146b-5p) was inversely correlated with NIS expression in all the cell lines where NIS was induced by selumetinib.

Hsa-let7f-5p was shown to be up-regulated in samples obtained by patients affected by thyroid cancer (FTC, PTC and ATC) [19]. Here, we found that hsa-let7f-5p was suppressed by selumetinib administration. Moreover, suppression of hsa-let7f-5p was accompanied by enhanced NIS transcript expression. Thus, our results support a possible role of hsa-let7f-5p in NIS regulation, as expected, according to a previous in-silico analysis [19]. Moreover, this is in line with a study that showed that hsa-let7f-5p exerts a tumor suppressor role by reducing cell proliferation and inducing thyroid differentiation markers in TC [24]. However, the determination of the specific role of hsa-let7f-5p in the complex network of NIS regulation needs further investigations.

So far, this study was characterized by experiments performed only in vitro in thyroid cancer cell lines; in vivo investigations are strongly needed in order to better understand the biological activity of selumetinib in the tumor environment. Interestingly, it has been shown that selumetinib is capable of restoring the expression of MHC Class I (major histocompatibility complex Class I) in papillary thyroid cancer and favors the immune response [43]. Thus, it would be important to detect the mechanisms induced by selumetinib in the tumor stroma/immune cells of FTC and ATC and the possible modulation of the previously mentioned miRNAs in those cells and their potential to modulate the expression of the MHC Class I.

## 4. Material and Methods

### 4.1. Cell Lines

Four cell lines with various histopathological backgrounds and mutation statuses were used. Two originated from papillary TCs (TPC1 and BCPAP), one from an anaplastic TC (C643) and one represent cells from an undifferentiated TC (8505C) [44]. The mutation status is as follows: TPC1, *Ret/PTC*; C643, *HRAS* and *TP53*; BCPAP, *BRAFV600E*; and 8505C, *BRAFV600E* and *TP53.*

TPC1 [45] and C643 [46] cells were kindly provided by Prof. A. Zielke (Diakonie-Klinikum Stuttgart, Stuttgart, Germany), whereas BCPAP [47] and 8505C [44] cell lines were purchased from the DSMZ (Leibnitz Institute DSMZ-German Collection of Microorganisms and cell Cultures, Braunschweig, Germany).

### 4.2. Cell Culture

All cell lines were grown in RPMI 1640 (Biochrom, Berlin, Germany) supplemented with 10% fetal bovine serum (FBS, Biochrom) and 10 U/mL penicillin and 100 µg/mL streptomycin (Biochrom) under standard conditions (37 °C, 5% CO_2_). They were routinely tested for Mycoplasma contamination.

### 4.3. Drug Preparation

Selumetinib (AZD6244) was obtained from Selleck Chemicals (Houston, TX, USA), dissolved in DMSO (WAK Chemicals, Steinbach, Germany) (20 mM) and kept at −80 °C. Working solutions were prepared with medium. Actinomycin was purchased by SIGMA-Aldrich (St. Louis, MO, USA).

### 4.4. Analysis of Cell Viability

Selumetinib-induced effects on cell viability were assessed by MTT-assay. Briefly, cells were seeded to 96-well culture plates (10^4^ cells/well) 24 h before treatment with selumetinib (0.1–100 µM). Incubation was continued for six days with changing the medium after three days. After 72 h and 144 h, metabolically active cells were determined by MTT colorimetric assay. Optical density was determined at 570 nm with a reference filter of 630 nm (Emax microplate reader, Molecular Devices, Munich, Germany). Experiments were performed three times in triplicate.

Data analysis: The raw data (optical density) were analysed using the standard Sigmoid Emax model (i.e., Hill model) to model the effect (E) as a function of the dose (d) and to calculate the median lethal dose (ED50). For all pair wise comparisons of the response at different doses with that of the control group, we used generalized least squares (GLS) estimation and *p*-values. Each set of comparisons was calculated as described by Hothorn et al. 2008. All statistical analyses were performed using the R program (3.0.0, Bell Laboratories, Murray Hill, NJ, USA) for statistical computing.

### 4.5. RNA Isolation and Quantitative Real Time RT-PCR

Cells were seeded in 25 cm^2^ cell culture flasks (5 × 10^5^ cells/flask) and treated with selumetinib (1µM and 10 µM) for up to 48 h. Total RNA, including short RNA was isolated by use of miRNeasy Mini Kit (Qiagen, Hilden, Germany) according to the manufacturer’s instructions.

miRNA-enriched RNA was reverse transcribed with miScript II RT Kit (Qiagen). cDNA was amplified with miScript SYBR Green PCR Kit (Qiagen) using a CFX96 cycler (Biorad, Munich, Germany) and hsa-let-7f-5p (MS00006489), hsa-miR-146b-5p (MS00003542) and hsa-miR-146b-3p (MS00008722) miScript Primer Assays (Qiagen, Hilden, Germany). RNU6B (MS00029204), also from Qiagen, was amplified as internal control miRNA.

For the amplification of NIS, RNA lysates were reverse transcribed using the iScript cDNA Syntesis Kit (Bio-Rad, Munich, Germany). PCR was run with the SsoFast Eva Green Supermix (BioRad) on CFX96 cycler. GAPDH was amplified as reference gene. Primer Assays for NIS (PPH10926A) and GAPDH (QT01192646, Qiagen, Hilden, Germany) were purchased from Qiagen.

Data Analysis: Results were analysed using CFX Manager (BioRad) and Rest 2008. Significance was calculated using the *t*-test for paired samples. *p* < 0.01 and *p* < 0.05 were regarded as significant.

### 4.6. Protein Isolation and Western Blotting

Cells were seeded in 75 cm^2^ cell culture flasks (1.5 × 10^6^ cells/flask) and incubated with or without selumetinib (1 µM and 10 µM) for 72 h. Then, cells were trypsinized, the suspension was collected and lysed with RIPA (Santa Cruz, Heidelberg, Germany) containing protease and phosphatase inhibitors (71 µL 7× protease cocktail and 50 µL 10× phosphatase cocktail (Roche, Basel, Switzerland) per 500 µL RIPA buffer). Protein content was determined by BCA-assay (Pierce, Rockford, IL, USA). Samples adjusted to 50 µg were separated on SDS-PAGE (NuPAGE Novex 4–12% Bis-Tris gels, NuPage MOPS running buffer (Invitrogen by Life Technologies, Carlsbad, CA, USA) and transferred to nitrocellulose (Amersham, Piscataway, NJ, USA). Membranes were probed using anti-human sodium iodide symporter (hNIS), clone FP5A (1:500) (Thermofisher Scientific, Fremont, CA, USA) as primary antibody. HRP conjugated secondary antibodies were from SIGMA-Aldrich. Visualization was performed by ECL western blotting reagent (Amersham) and using an image capture and analysis system (Fusion, PeqLab, Erlangen, Germany). Equal loading was verified by anti-GAPDH (Abcam 9485. Working dilution 1:2500) (Abcam, Cambridge, MA, USA). The protein amount was densitometrically quantified by the use of Bio-1D Software (Peqlab GmbH, Erlangen, Germany).

### 4.7. In Vitro Radioiodine Uptake

For the analysis of radioiodine uptake, the cells were seeded in 6-well plates at a density of 4 × 10^5^ cells/well. Immediately after treatment with 10 µM selumetinib, 1 Mbq I-131 was added to the cells and the plates were incubated for 48 h. Subsequently, the supernatant was collected, the cells were washed with PBS and trypsinized for 5 min. The suspension was rinsed with 3 mL PBS, collected and centrifuged at 900 rpm for 5 min. The supernatant was discarded and the pellet was suspended in 5 mL PBS. The suspension was once more centrifuged. The supernatant was discarded and the cells were processed into a Gamma counter for the measurement of the retained radioactive I-131. The untreated cells were used as control for the measurement.

## 5. Conclusions

The present study demonstrates for the first time that selumetinib mediates the restoration of NIS via the suppression of its related miRNAs in thyroid cancer cell lines. The observed mechanism was not correlated with the BRAF status of the model used for the study. The restoration of NIS mediated by targeting miRNA inhibition offered to the treated cells a greater amount of NIS transcript, facilitating the turn-over of the sodium iodide symporter and maintaining it in an active status that favours cell differentiation. Further studies are needed to clarify the specific role of hsa-miR-146b-5p and hsa-let7f-5p mediating NIS expression and how their modulation could possibly contribute to the restoration of NIS functionality in advanced radioiodine refractory thyroid cancer.

## Figures and Tables

**Table 1:** *p*-Values showing that selumetinib treatment was significant at all concentrations for all four cells lines even for the lowest used concentration. *p* < 0.05 regarded as significant.

Line	Contrast	Estimate	Lower	Upper	*p*
TPC1	0.1 vs. control	−0.07	−0.12	−0.01	0.02
TPC1	1.0 vs. control	−0.10	−0.16	−0.04	0.00
TPC1	10 vs. control	−0.14	−0.22	−0.07	0.00
TPC1	100 vs. control	−0.21	−0.30	−0.13	0.00
C643	0.1 vs. control	−0.12	−0.24	−0.00	0.04
C643	1.0 vs. control	−0.34	−0.48	−0.20	0.00
C643	10 vs. control	−0.66	−0.87	−0.46	0.00
C643	100 vs. control	−1.01	−1.32	−0.70	0.00
BCPAP	0.1 vs. control	−0.12	−0.22	−0.02	0.02
BCPAP	1.0 vs. control	−0.17	−0.29	−0.06	0.00
BCPAP	10 vs. control	−0.19	−0.32	−0.07	0.00
BCPAP	100 vs. control	−0.29	−0.44	−0.14	0.00
8505C	0.1 vs. control	−0.19	−0.23	−0.15	0.00
8505C	1.0 vs. control	−0.30	−0.36	−0.24	0.00
8505C	10 vs. control	−0.33	−0.40	−0.26	0.00
8505C	100 vs. control	−0.34	−0.42	−0.28	0.00

**Table 2:** Expression of miRNAs and SLC5A5 transcript in TPC1, C643, BCPAP and 8505C cells after treatment with 10 µM Selumetinib. For final representation, PCR data (fold change compared to untreated sample) were classified as follows: ↓ < 0.5; ↑ > 10; ±no variations/stable; n.d. not detectable.

	hsa-let7f-5p	hsa-miR-146b-5p	hsa-miR-146b-3p	SLC5A5
TPC1	↓	↓	n.d.	↑
C643	↑	±/↑	↑	±
BCPAP	↓	↓	↓	↑
8505C	±/↑	↓	±/↑	↑
